# Birth conditions nutritional status in childhood associated with cardiometabolic risk factors at 30 years of age: a cohort study

**DOI:** 10.1590/0102-311XEN215522

**Published:** 2023-06-26

**Authors:** Vânia Pereira Oliveira, Mariane da Silva Dias, Natália Peixoto Lima, Bernardo Lessa Horta

**Affiliations:** 1 Programa de Pós-graduação em Epidemiologia, Universidade Federal de Pelotas, Pelotas, Brasil.; 2 Universidade Federal do Rio Grande, Rio Grande, Brasil.; 3 Departamento de Medicina Social, Universidade Federal de Pelotas, Pelotas, Brasil.

**Keywords:** Nutritional Status, Growth, Cardiometabolic Risk Factors, Cohort Studies, Estado Nutricional, Crescimento, Fatores de Risco Cardiometabólico, Estudos de Coortes, Estado Nutricional, Crecimiento, Factores de Riesgo Cardiometabólico, Estudios de Cohortes

## Abstract

This study aimed to assess the association of birth conditions, nutritional status, and childhood growth with cardiometabolic risk factors at 30 years of age. We also evaluated whether body mass index (BMI) at 30 years mediated the association of weight gain in childhood with cardiometabolic risk factors. This is a prospective cohort study that included all live births in 1982 in hospitals in the city of Pelotas, Rio Grande do Sul State, Brazil, whose families lived in the urban area. Mothers were interviewed at birth, and participants were followed at different ages. For our analyses, we used data on weight and height collected at birth, 2 and 4 years and cardiovascular risk factors at 30 years. Multiple linear regressions were performed to obtain adjusted coefficients and G-formula for mediation analysis. Relative weight gain in childhood, despite the age, was positively related to mean arterial pressure, whereas relative weight gain in late childhood was positively associated with carotid intima-media thickness, pulse wave velocity, triglycerides, non-HDL cholesterol, plasma glucose, and C-reactive protein. BMI in adulthood captured the total effect of relative weight gain in the period between 2 and 4 years on carotid intima-media thickness, triglycerides, non-HDL cholesterol, and C-reactive protein. Our findings reinforce the evidence that rapid relative weight gain after 2 years of age may have long-term consequences on the risk of metabolic and cardiovascular disorders.

## Resumo

O objetivo deste estudo foi avaliar a associação das condições de nascimento, do estado nutricional e do crescimento infantil com fatores de risco cardiometabólicos aos 30 anos de idade. Também foi verificado se o índice de massa corporal (IMC) aos 30 anos mediava a associação entre o ganho de peso na infância e fatores de risco cardiometabólicos. Trata-se de um estudo de coorte prospectivo que incluiu todos os nascidos vivos em 1982 em hospitais da cidade de Pelotas, Rio Grande do Sul, Brasil, residentes da área urbana. As mães foram entrevistadas no parto e os participantes foram acompanhados em diferentes idades. Para as análises, foram utilizados os dados de peso e altura coletados no nascimento e aos 2 e 4 anos de idade e fatores de risco cardiovascular aos 30 anos. Regressões lineares múltiplas foram realizadas para a obtenção de coeficientes ajustados e G-fórmula para a análise de mediação. O ganho de peso relativo na infância, apesar da idade, está positivamente associado à pressão arterial média, enquanto o ganho de peso relativo tardio na infância está positivamente associado à espessura médio-intimal da artéria carótida, à velocidade da onda de pulso, aos triglicerídeos, ao colesterol não-HDL, à glicose plasmática e à proteína C reativa. O IMC na idade adulta capturou o efeito total do ganho de peso relativo entre 2 e 4 anos sobre a espessura médio-intimal da carótida, os triglicerídeos, o colesterol não-HDL e a proteína C reativa. Estes achados reforçam a evidência de que o rápido ganho de peso relativo após os 2 anos de idade pode ter consequências a longo prazo sobre o risco de distúrbios metabólicos e cardiovasculares.

## Resumen

El objetivo de este estudio fue evaluar la asociación de las condiciones de nacimiento, estado nutricional y crecimiento infantil con factores de riesgo cardiometabólico a los 30 años de edad. También se verificó si el índice de masa corporal (IMC) a los 30 años mediaba la asociación entre el aumento de peso infantil y los factores de riesgo cardiometabólicos. Se trata de un estudio de cohorte prospectivo que incluyó todos los nacidos vivos en 1982 en hospitales de la ciudad de Pelotas, estado de Río Grande del Sur, Brasil, residentes del área urbana. Las madres fueron entrevistadas en el momento del parto y los participantes fueron seguidos a diferentes edades. Para los análisis, utilizamos los datos de peso y altura recopilados al nacer y a los 2 y 4 años de edad y los factores de riesgo cardiovascular a los 30 años. Se realizaron regresiones lineales múltiples para obtener coeficientes ajustados y la G-fórmula para el análisis de mediación. El aumento de peso relativo en la infancia, a pesar de la edad, se asocia positivamente con la presión arterial media, mientras que el aumento de peso relativo en la infancia tardía se asocia positivamente con el espesor de la íntima-media de la arteria carotídea, la velocidad de la onda del pulso, los triglicéridos, el colesterol no HDL, la glucosa plasmática y la proteína C reactiva. El IMC en adultos capturó el efecto completo del aumento de peso relativo a los 2 y 4 años sobre el espesor de la íntima-media carotídea, los triglicéridos, el colesterol no HDL y la proteína C reactiva. Estos hallazgos refuerzan la evidencia de que el rápido aumento de peso relativo después de los 2 años puede tener consecuencias a largo plazo sobre el riesgo de trastornos metabólicos y cardiovasculares.

## Introduction

Noncommunicable diseases are the leading cause of death worldwide, being responsible for about 40 million deaths every year ^1^. About 77% of deaths from these diseases occur in low- and middle-income countries ^1^. Evidence suggests that the development of noncommunicable diseases may be programmed by early life exposures, such as birth weight, nutritional status, and childhood growth ^2^ ^,^ ^3^ ^,^ ^4^ ^,^ ^5^ ^,^ ^6^.

Studies suggest that poor nutritional status in early childhood have a negative impact on human capital, whereas rapid growth increases the risk of noncommunicable diseases ^7^ ^,^ ^8^ ^,^ ^9^. However, most of the early studies on the long-term consequences of childhood growth have assessed the effect of weight gain ^10^. Because weight gain is related to linear growth and changes in soft tissue, it is important to disentangle the effect of linear growth from weight gain relative to linear growth. Moreover, it is also important to assess the impact of the timing of growth. It has been reported that growth during early childhood may be positively associated with performance in intelligence tests, and this association would be stronger for linear growth ^8^ ^,^ ^9^. On the other hand, growth during the middle of childhood would not impact human capital. Concerning noncommunicable diseases, evidence suggests that faster relative weight gain would be positively associated with an increased risk of obesity, coronary heart disease, type 2 diabetes, and hypertension ^8^ ^,^ ^11^ ^,^ ^12^ ^,^ ^13^.

Most studies on the long-lasting consequences of childhood growth have not evaluated the effect of the timing. Moreover, the effect of linear growth has not been disentangled from that of linear growth-independent weight gain. To the best of our knowledge, the mediating role of body mass index (BMI) in adulthood in the association of conditional relative weight gain during childhood with cardiometabolic risk factors in adulthood has not been evaluated. This study aimed to assess the association of birth conditions, nutritional status, and growth during childhood with cardiometabolic risk factors at 30 years of age, and to examine whether the association between relative weight gain and cardiometabolic risk factors was mediated by BMI in adulthood.

## Methodology

In 1982, the maternity hospitals located in Pelotas, a southern Brazilian city, were daily visited, and those live births whose families lived in the urban area of the city (N = 5,914) were examined and their mothers were interviewed soon after childbirth. These subjects have been prospectively followed up on several occasions, and further details on the study methodology have been published elsewhere ^14^ ^,^ ^15^.

In 1984 and 1986, all households located in the urban area of the city were visited in search of the cohort members, and 5,161 and 4,979 individuals were evaluated, respectively. From June 2012 to February 2013, the research team tried to follow the whole cohort and the study participants were invited to visit the research clinic to be interviewed, examined, and to donate a random blood sample. In this study, pregnant women were excluded.

### Outcomes

At 30 years, the following cardiometabolic risk factors were assessed:

Blood pressure was measured twice on the left arm. The measurement was performed using an automatic device (Omron HEM 705C PINT; https://www.omron.com) with an adapted cuff for subjects with obesity, and the average of these measurements was used in the analyses. Average arterial pressure was estimated by: 1/3 systolic blood pressure + 2/3 diastolic blood pressure ^16^.

Carotid intima-media thickness was measured at the posterior wall of the right and left common carotid arteries in longitudinal planes using ultrasound imaging. Photographs of a 10mm-long section of the common carotid artery were taken, proximal to the carotid bulb. The Carotid Analyzer for Research (Medical Imaging Applications; http://www.mia-llc.com/) was used to analyze image data. The analyzer also calculated the average value of 90 measurements (frames) taken from the 10mm-long section studied.

Pulse wave velocity was assessed using the SphygmoCor system (Atcor Medical, Version 9.0; https://atcormedical.com/), which is a noninvasive device that measures the pulse wave velocity with a tonometric transducer.

Serum glucose was measured using the colorimetric enzyme assay with K082 Glucose Monoreagent kits (Bioclin; https://www.bioclin.com.br/).

High-sensitivity C-reactive protein was measured using the automated turbidimetry technique with a BS-380 (Shenzhen-Mindray Bio-Medical Electronics; https://www.mindray.com/en) chemistry analyzer.

Total cholesterol, HDL cholesterol, and triglycerides were processed via automated enzymatic colorimetric methods in a chemistry analyzer (BS-380, Shenzhen-Mindray Bio-Medical Electronics).

Women who used oral contraceptives were excluded from C-reactive protein analyses since oral contraceptives increase C-reactive protein levels ^17^. Those participants who were taking hypoglycemic agents, antihypertensive drugs or statins medicines were excluded from glucose, blood pressure, and lipid profile analyses, respectively.

### Early-life exposures

Birth weight was assessed by the hospital staff using pediatric scales (Filizola; https://www.oswaldofilizola.com.br/) that were periodically calibrated by the research team. Gestational age was estimated based on the date of the last menstrual period, and birth weight for gestational age z-scores were assessed using the Williams reference population ^18^.

At 2 (1984) and 4 years of age (1986), cohort members were weighted to the nearest 0.1kg using portable calibrated scales (CMS Weighing Equipment Ltd., United Kingdom) and portable stadiometers were used to assess length/height to the nearest 0.1cm. Weight-for-length/height, BMI-for-age, and height/length-for-age z-scores according to age and sex were estimated using the World Health Organization (WHO) growth standards ^19^.

Conditional growth was assessed by regressing current size (weight or length/height) on earlier measures of weight and length/height, and standardized residuals were derived by sex. Conditional variables express how a child deviates from its expected height or weight based on its previous measures and the growth of the same sex from the studied population. At each time point, the conditional variable represents growth during a time interval. For example, conditional relative weight at 2 years of age represents the relative weight gain from birth to 2 years of age; similarly, the conditional variable at 4 years of age represents the height or relative weight gain from 2 to 4 years of age. To estimate conditional height, the current length/height was regressed on previous weight and length. Therefore, conditional length at 2 years of age was estimated by regressing length-for-age z-scores at 2 years of age on birth weight. In contrast, conditional relative weight was estimated from length/height at that age and previous measures of length/height and weight. Thus, conditional relative weight at 2 years of age was derived by regressing weight at 2 years of age on birth weight and length at 2 years of age ^8^ ^,^ ^9^.

### Confounders and mediator

Family income at birth assessed the total income earned by the family members in the month before the interview. Maternal schooling at birth was assessed in complete years of schooling. Maternal skin color was evaluated by the interviewer in the perinatal study and categorized into two groups (white/non-white). Mothers who reported any smoking during pregnancy were considered as smokers. Maternal height was measured at the hospital in the perinatal study, using portable stadiometers. Pre-pregnancy weight was obtained from the antenatal care card or reported by the mother. Information on breastfeeding duration and age at introduction of weaning foods was collected in the 1984 and 1986 visits. The information closest to the age of weaning was used to minimize recall bias.

BMI at 30 years, a possible mediator, was assessed in the 2012/2013 follow-up visit. Weight was measured to the nearest 0.1kg using a scale coupled to BodPod (COSMED; https://www.cosmed.com) and height was measured with a portable stadiometer (SECA 240; https://www.seca.com). The BMI was calculated by dividing the weight by the square height (kg/m^2^).

### Statistical analysis

Statistical analysis was performed using Stata software package, version 16 (https://www.stata.com). As the distribution of triglycerides and C-reactive protein were clearly asymmetric, these variables were log-transformed. Analysis of variance (ANOVA) was used to compare means and multiple linear regression to obtain estimates that were adjusted for confounders (maternal education, family income at birth, maternal skin color, maternal smoking during pregnancy, parity, maternal pre-pregnancy BMI, gestational age, maternal age). Estimates on the associations of nutritional status in childhood and conditional growth were further adjusted to breastfeeding duration. Furthermore, for conditional length gain from birth to 2 years of age, analyses were also adjusted for birth weight according to gestational age z-score. For conditional relative weight gain from birth to 2 years of age, analyses were controlled for birth weight and conditional length gain from 0 to 2 years of age. For conditional length gain from 2 to 4 years of age, birth weight and conditional variables from birth to 2 years of age were included in the regression model. For relative weight gain from 2 to 4 years of age, length gain for this period was further included in the model. Conditional relative weight and conditional height variables are not correlated to each other and, hence, they were included together in linear regression models without collinearity concerns ^8^ ^,^ ^9^.

The covariates included in the models were selected a priori following a conceptual model based on the literature. In addition to verifying the correlation between the variables included in the models, the variance inflation factor (VIF) was also verified for each of the regression models and there was a low possibility of multicollinearity in the models. For ordinal variables, comparisons were based on tests of heterogeneity and linear trend, and the one with the lowest p-value was presented. Residual analyses were performed and presented as Supplementary Material (https://cadernos.ensp.fiocruz.br/static//arquivo/suppl-e00215522_7082.pdf). Generally, the points are randomly distributed, complying with the assumptions of linearity, normality, and homoscedasticity.

Mediation analysis was conducted using G-formula to decompose the total effect into natural direct and indirect effects of conditional relative weight gain from 2 to 4 years of age on metabolic cardiovascular risk factors at age 30. Standard errors for mediation analyses were calculated using bootstrapping with 10,000 simulations. Separate models were fitted for each outcome (triglycerides, non-HDL cholesterol, plasma glucose, C-reactive protein, mean arterial pressure, carotid intima-media thickness, pulse wave velocity, and glomerular filtration rate) and mediator (BMI at 30 years of age). All models were adjusted for base confounders (family income; maternal schooling, age at birth, skin color, and smoking during pregnancy; parity; pre-pregnancy BMI; birth weight for gestational age; breastfeeding duration; conditional length 0-2 years; relative conditional weight 0-2 years; and conditional height 2-4 years) and post-confounders (family income at last visit). Before conducting the mediation analyses, it was assessed whether body mass index at adulthood was modifying the associations.

### Ethical standards

The current study was conducted in accordance with the *Declaration of Helsinki*, and all procedures involving human subjects were approved by Research Ethics Committee of the Faculty of Medicine, Federal University of Pelotas (protocol n. 16/12).

## Results

In the 30-year follow-up of the 1982 Pelotas birth cohort, 3,701 subjects were evaluated, resulting in a follow-up rate of 68.1% when combined with the 325 deaths among the cohort participants. Information on at least one exposure and outcome was available for 3,619 individuals. For the conditional growth analysis, information on birth weight, gestational age, and nutritional status at 2 and 4 years of age was available for 2,479 subjects. Table 1 shows that about seven of every ten subjects included in the analyses were born in families with an income ≤ 3 minimum wages, 7.2% had low birth weight, and 5.7% were preterm. Mean arterial pressure at 30 years was 90.4mmHg.

Tables 2, 3, 4, and 5 shows the crude estimates for the associations of birth conditions and nutritional status in early childhood with metabolic cardiovascular risk factors.

**Table 1:** Characteristics of the studied population.

Characteristics	Sample (N = 3,619) *	
	n	%
Gender	3,619	
Man	1,773	49.0
Woman	1,846	51.0
Maternal schooling (years)	3,615	
0-4	1,166	32.3
5-8	1,553	43.0
9-11	398	11.0
≥ 12	498	13.8
Maternal skin color	3,618	
White	2,970	82.1
Non-White	648	17.9
Family income at birth (minimum wages)	3,603	
≤ 1	714	19.8
1.1-3	1,782	49.5
3.1-6	702	19.5
6.1-10	217	6.0
> 10	188	5.2
Gestational age (weeks)	2,916	
< 37	165	5.7
37-38	643	22.0
> 39	2,108	72.3
Birth weight (g)	3,619	
< 2,500	261	7.2
2,500-2,999	860	23.8
3,000-3,499	1,364	37.7
≥ 3,500	1,134	31.3
	n	Mean (SD)
Mean arterial pressure (mmHg)	3,535	90.4 (10.03)
Carotid intima-media thickness (µm)	2,997	5,832.7 (231.6)
Non-HDL cholesterol (mg/dL)	3,515	132.4 (35.9)
Glomerular filtration rate (mL/minutes/per 1.73m^2^)	3,530	129.5 (31.4)
Pulse wave velocity (m/s)	1,572	6.4 (1.1)
Plasma glucose (mg/dL)	3,518	89.2 (24.7)
	n	Geometric mean (IQR)
Triglycerides (mg/dL)	3,515	4.55 (4.20; 4.96)
C-reactive protein (mg/dL)	2,340	0.33 (-0.51; 1.22)

IQR: interquartile range; SD: standard deviation.

* Individuals included in the analyses with information for at least one exposure of interest and outcome assessed at age 30.

**Table 2:** Mean arterial pressure, carotid intima-media thickness, pulse wave velocity, and glomerular filtration rate at 30 years, according to birth conditions and nutritional status. Crude analyses.

	Mean arterial pressure		Carotid intima-media thickness		Pulse wave velocity	
	n	Mean (SD)	n	Mean (SD)	n	Mean (SD)
Gestational age (weeks)	2,916	p = 0.5073	2,448	p = 0.5831	1,251	p = 0.1965
< 37	165	90.79 (10.23)	136	5,833.8 (229.99)	75	6.21 (1.01)
37-38	643	91.15 (10.62)	548	5,840.0 (239.09)	278	6.41 (1.04)
> 39	2,108	90.62 (10.04)	1,764	5,828.7 (219.9)	898	6.44 (1.13)
Birth weight according to gestational age (z-score)	2,915	p = 0.5269	2,447	p = 0.1316	1,251	p = 0.7726
< -1.28	417	90.91 (10.51)	345	5,837.5 (234.92)	179	6.38 (1.06)
-1.28/0	1,293	90.93 (10.24)	1,070	5,821.2 (214.94)	540	6.44 (1.05)
> 0	1,205	90.50 (10.00)	1,032	5,840.3 (231.26)	532	6.42 (1.16)
Birth weight (g)	3,619	p = 0.7971	3,011	p = 0.2057	1,576	p = 0.9805
< 2,500	261	90.83 (10.76)	203	5,829.0 (243.17)	119	6.38 (0.98)
2,500-2,999	860	90.76 (10.16)	684	5,828.5 (229.21)	375	6.43 (1.07)
3,000-3,499	1,364	90.57 (10.36)	1,150	5,825.4 (232.02)	583	6.42 (1.10)
≥ 3,500	1,134	90.98 (10.19)	974	5,845.9 (231.14)	499	6.43 (1.14)
Height/length-for-age z-scores - 2 years	3,319	p = 0.1210	2,759	p = 0.5388	1,459	p = 0.0934
≤ -2	428	90.50 (10.38)	356	5,831.89 (217.25)	206	6.49 (1.02)
-2/-1	825	90.68 (10.32)	668	5,840.97 (268.38)	355	6.37 (1.06)
-1/1	1,813	92.23 (10.16)	1,522	5,827.67 (220.14)	790	6.40 (1.14)
≥ 1	253	92.23 (11.28)	213	5,845.08 (221.86)	108	6.65 (0.99)
Height/length-for-age z-scores - 4 years	3,251	p = 0.0004	2,709	p = 0.1214	1,423	p = 0.0109
≤ -2	345	90.54 (10.29)	278	5,838.42 (246.30)	158	6.47 (1.03)
-2/-1	846	89.93 (10.31)	701	5,827.85 (235.95)	377	6.36 (1.03)
-1/1	1,842	90.97 (10.22)	1,548	5,830.08 (225.64)	799	6.42 (1.14)
≥ 1	218	93.15 (10.48)	182	5,871.69 (244.81)	89	6.78 (1.08)
Weight-for-length/height z-scores - 2 years	3,318	p = 0.2194	2,758	p = 0.1143	1,459	p = 0.3320
≤ -1	168	90.83 (8.82)	138	5,821.84 (205.31)	71	6.56 (1.23)
-1/1	2,060	90.58 (10.63)	1,714	5,825.55 (227.12)	913	6.40 (1.09)
1/2	848	90.75 (10.03)	703	5,848.11 (249.39)	371	6.45 (1.02)
≥ 2	242	92.05 (9.63)	203	5,848.48 (232.68)	104	6.56 (1.32)
Weight-for-length/height z-scores - 4 years	3,249	p < 0.0001	2,708	p = 0.0019	1,423	p = 0.0021
≤ -1	157	90.69 (9.56)	125	5,824.25 (235.29)	70	6.48 (1.09)
-1/1	2,114	90.16 (10.07)	1,782	5,821.95 (218.50)	903	6.37 (1.07)
1/2	757	91.88 (10.77)	618	5,857.56 (252.48)	339	6.45 (1.06)
≥ 2	221	93.35 (10.65)	183	5,866.05 (274.11)	111	6.79 (1.41)

SD: standard deviation.

Note: analysis of variance (ANOVA).

**Table 3:** Triglycerides, non-HDL cholesterol, plasma glucose, and C-reactive protein at 30 years, according to birth conditions and nutritional status. Crude analyses.

	Triglycerides		Non-HDL cholesterol		Plasma glucose		C-reactive protein	
	n	Mean (SD)	n	Mean (SD)	n	Mean (SD)	n	Mean (SD)
Gestational age (weeks)	2,854	p = 0.5866	2,854	p = 0.3874	2,844	p = 0.3246	1,888	p = 0.6822
< 37	162	4.58 (0.52)	162	130.14 (34.75)	161	86.48 (13.22)	111	0.44 (1.42)
37-38	630	4.62 (0.59)	630	133.95 (42.54)	627	89.49 (30.64)	398	0.32 (1.29)
> 39	2,062	4.62 (0.55)	2,062	132.14 (34.19)	2,056	89.82 (20.25)	1,379	0.35 (1.27)
Birth weight according to gestational age (z-score)	2,853	p = 0.6045	2,853	p = 0.7484	2,843	p = 0.4131	1,888	p = 0.9991
< -1.28	406	4.64 (0.57)	406	133.36 (35.67)	406	89.46 (18.74)	283	0.34 (1.29)
-1.28/0	1,268	4.61 (0.56)	1,268	132.66 (37.12)	1,265	89.27 (24.62)	827	0.35 (1.24)
> 0	1,179	4.62 (0.55)	1,179	131.89 (35.45)	1,172	88.17 (21.66)	778	0.35 (1.33)
Birth weight (g)	3,540	p = 0.0534	3,540	p = 0.1404	3,527	p = 0.1070	2,347	p = 0.6467
< 2,500	251	4.63 (0.55)	251	128.69 (30.92)	250	87.53 (14.86)	161	0.38 (1.37)
2,500-2,999	842	4.63 (0.59)	842	134.60 (41.82)	839	90.92 (32.99)	523	0.39 (1.31)
3,000-3,499	1,331	4.58 (0.55)	1,331	132.23 (35.5)	1,326	88.58 (21.91)	878	0.32 (1.28)
≥ 3,500	1,116	4.64 (0.56)	1,116	132.43 (35.55)	1,112	89.13 (21.94)	785	0.31 (1.26)
Height/length-for-age z-scores - 2 years	3,242	p = 0.1097	3,242	p = 0.6835	3,231	p = 0.0074	2,146	p = 0.9575
≤ -2	418	4.58 (0.58)	418	132.42 (39.41)	418	92.28 (37.39)	310	0.36 (1.28)
-2/-1	803	4.61 (0.55)	803	133.84 (36.62)	802	88.92 (19.10)	553	0.34 (1.28)
-1/1	1,772	4.62 (0.55)	1,772	132.06 (36.14)	1,762	87.98 (22.19)	1,120	0.34 (1.26)
≥ 1	249	4.69 (0.60)	249	133.67 (38.88)	249	91.08 (31.58)	163	0.29 (1.33)
Height/length-for-age z-scores - 4 years	3,177	p < 0.0001	3,177	p = 0.0169	3,166	p = 0.0560	2,109	p = 0.6670
≤ -2	337	4.51 (0.55)	337	130.18 (36.44)	337	89.95 (26.10)	235	0.32 (1.27)
-2/-1	826	4.62 (0.56)	826	133.61 (37.14)	823	89.46 (20.94)	543	0.29 (1.27)
-1/1	1,799	4.62 (0.55)	1,799	131.74 (35.85)	1,791	88.26 (22.50)	1,183	0.36 (1.29)
≥ 1	215	4.76 (0.63)	215	139.28 (41.33)	215	92.64 (37.36)	148	0.42 (1.27)
Weight-for-length/height z-scores - 2 years	3,241	p = 0.2725	3,241	p = 0.6751	3230	p = 0.2533	2,146	p = 0.4385
≤ -1	165	4.64 (0.59)	165	134.26 (37.98)	165	90.33 (21.61)	115	0.37 (1.37)
-1/1	2,010	4.61 (0.56)	2,010	132.10 (38.12)	2,003	89.54 (27.35)	1,303	0.33 (1.27)
1/2	827	4.60 (0.56)	827	133.12 (34.46)	825	88.03 (21.30)	558	0.31 (1.29)
≥ 2	239	4.67 (0.55)	239	134.57 (33.88)	237	87.08 (14.00)	170	0.49 (1.18)
Weight-for-length/height z-scores - 4 years	3,175	p = 0.0035	3,175	p = 0.1237	3,164	p = 0.3857	2,108	p = 0.0037
≤ -1	153	4.66 (0.55)	153	132.22 (35.14)	152	89.01 (18.63)	111	0.34 (1.46)
-1/1	2,066	4.59 (0.55)	2,066	131.50 (36.41)	2,060	88.63 (22.92)	1,310	0.26 (1.28)
1/2	734	4.65 (0.55)	734	135.19 (36.83)	731	89.44 (23.38)	521	0.49 (1.23)
≥ 2	222	4.70 (0.64)	222	133.78 (39.43)	221	91.43 (34.36)	166	0.48 (1.25)

SD: standard deviation.

Note: analysis of variance (ANOVA).

**Table 4:** Mean arterial pressure, carotid intima-media thickness, pulse wave velocity, and glomerular filtration rate at 30 years, according to conditional growth in childhood. Crude analyses.

	Mean arterial pressure		Carotid intima-media thickness		Pulse wave velocity	
	n	Mean (SD)	n	Mean (SD)	n	Mean (SD)
Conditional length at age 0-2 years (z-score)	2,668	p = 0.0318	2,240	p = 0.7240	1,155	p = 0.4530
≤ 0	1,336	90.31 (10.08)	1,117	5,829.36 (223.91)	595	6.40 (1.10)
0/1	525	90.37 (10.48)	429	5,823.51 (219.51)	196	6.39 (1.11)
1/2	423	91.38 (9.67)	360	5,840.88 (219.97)	188	6.46 (1.02)
≥ 2	394	91.78 (10.68)	334	5,834.92 (244.68)	176	6.54 (1.19)
Conditional relative weight at age 0-2 years (z-score)	2,667	p = 0.0004	2,239	p = 0.3999	1,155	p = 0.0717
≤ 0	1,390	90.78 (10.37)	1,169	5,823.61 (205.14)	608	6.43 (1.12)
0/1	490	89.49 (9.79)	407	5,833.81 (263.62)	201	6.31 (1.05)
1/2	396	90.33 (10.18)	334	5,840. 84 (236.67)	165	6.39 (0.94)
≥ 2	391	92.38 (9.88)	329	5,843.42 (232.80)	181	6.60 (1.23)
Conditional height at age 2-4 years (z-score)	2,481	p < 0.0001	2,078	p = 0.5351	1,070	p = 0.0956
≤ 0	1,247	90.12 (10.31)	1,038	5,828.59 (242.67)	552	6.43 (1.13)
0/1	487	90.12 (9.67)	406	5,829.02 (200.62)	195	6.41 (0.93)
1/2	365	91.83 (10.59)	308	5,839.49 (218.01)	145	6.29 (1.11)
≥ 2	382	92.88 (9.83)	326	5,848.10 (221.55)	178	6.59 (1.20)
Conditional relative weight at age 2-4 years (z-score)	2,479	p < 0.0001	2,077	p = 0.0251	1,070	p = 0.0621
≤ 0	1,318	89.99 (10.04)	1,103	5,822.19 (223.44)	542	6.36 (1.12)
0/1	519	90.94 (9.94)	438	5,840.75 (232.73)	234	6.45 (1.02)
1/2	323	91.66 (9.83)	268	5,832.58 (199.83)	140	6.47 (1.10)
≥ 2	319	93.06 (11.26)	268	5,868.11 (260.97)	154	6.63 (1.20)

SD: standard deviation.

Note: analysis of variance (ANOVA).

**Table 5:** Triglycerides, non-HDL cholesterol, plasma glucose, and C-reactive protein at 30 years, according to conditional growth in childhood. Crude analyses.

	Triglycerides		Non-HDL cholesterol		Plasma glucose		C-reactive protein	
	n	Mean (SD)	n	Mean (SD)	n	Mean (SD)	n	Mean (SD)
Conditional length at age 0-2 years (z-score)	2,607	p = 0.0511	2,607	p = 0.8541	2,599	p = 0.9390	1,723	p = 0.2530
≤ 0	1,306	4.59 (0.55)	1,306	132.71 (35.64)	1,303	88.68 (20.48)	906	0.37 (1.28)
0/1	503	4.62 (0.56)	503	131.32 (40.04)	502	88.07 (27.19)	309	0.23 (1.30)
1/2	411	4.64 (0.56)	411	133.30 (34.86)	407	88.96 (20.57)	260	0.36 (1.22)
≥ 2	387	4.68 (0.55)	387	132.64 (35.88)	387	88.47 (25.30)	248	0.42 (1.30)
Conditional relative weight at age 0-2 years (z-score)	2,606	p = 0.1533	2,606	p = 0.3989	2,598	p = 0.4288	1,723	p = 0.0988
≤ 0	1,360	4.63 (0.56)	1,360	132.95 (38.66)	1,354	89.27 (25.57)	890	0.37 (1.27)
0/1	477	4.58 (0.55)	477	130.32 (34.50)	475	88.07 (20.38)	299	0.23 (1.28)
1/2	384	4.60 (0.51)	384	131.89 (32.95)	384	87.55 (20.29)	257	0.29 (1.32)
≥ 2	385	4.66 (0.59)	385	134.29 (33.77)	385	87.78 (15.72)	277	0.48 (1.23)
Conditional height at age 2-4 years (z-score)	2,423	p = 0.0864	2,423	p = 0.4015	2,416	p = 0.4490	1,601	p = 0.0317
≤ 0	1220	4.59 (0.54)	1,220	131.46 (34.11)	1,216	87.97 (20.97)	754	0.32 (1.28)
0/1	473	4.62 (0.59)	473	134.17 (43.48)	471	89.63 (31.28)	327	0.23 (1.22)
1/2	359	4.66 (0.56)	359	132.09 (35.02)	359	88.10 (15.76)	258	0.42 (1.24)
≥ 2	371	4.66 (0.55)	371	134.31 (35.87)	370	89.57 (23.48)	262	0.53 (1.34)
Conditional relative weight at age 2-4 years (z-score)	2,421	p = 0.0064	2,421	p = 0.0345	2,414	p = 0.0071	1,600	p = 0.0015
≤ 0	1,287	4.59 (0.54)	1,287	131.13 (36.52)	1,284	87.58 (22.48)	797	0.24 (1.30)
0/1	505	4.60 (0.54)	505	131.44 (33.28)	503	88.47 (17.19)	342	0.44 (1.22)
1/2	315	4.67 (0.57)	315	135.37 (35.94)	314	88.42 (17.78)	220	0.36 (1.30)
≥ 2	314	4.70 (0.61)	314	136.85 (41.33)	313	92.63 (35.00)	241	0.58 (1.22)

SD: standard deviation.

Note: analysis of variance (ANOVA).

Table 6 shows that after controlling for confounding variables, both birth weight and birth weight, according to gestational age, were negatively associated with mean arterial pressure. Stunting at 2 and 4 years old was associated with higher mean arterial pressure. Height for age z-score at 2 and 4 years old was also associated with pulse wave velocity, but the pattern of association was unclear. Weight-for-height z-score at 2 years old was not associated with arterial pressure, carotid intima-media thickness, pulse wave velocity, whereas at 4 years of age a positive association with mean arterial pressure, carotid intima-media thickness, and pulse wave velocity was observed.

**Table 6:** Coefficients estimated by multiple linear regression for mean arterial pressure, carotid intima-media thickness, and pulse wave velocity at 30 years, according to birth conditions and nutritional status.

	Adjusted regression coefficient (95%CI)		
	Mean arterial pressure (mmHg) *	Carotid intima-media thickness (µm)	Pulse wave velocity (m/s)
Gestational age (weeks) **	p = 0.192 *** [n = 2,831]	p = 0.433 *** [n = 2,448]	p = 0.125 *** [n = 1,251]
> 39	Reference (0)	Reference (0)	Reference (0)
< 37	0.50 (-1.08; 2.04)	4.92 (-34.38; 44.21)	-0.23 (-0.48; 0.03)
37-38	0.60 (-0.26; 1.46)	10.80 (-10.80; 32.40)	-0.04 (-0.19; 0.11)
Birth weight according to gestational age (z-score) **	p = 0.012 * [n = 2,830]	p = 0.1375 ^#^ [n = 2,447]	p = 0.564 *** [n = 1,251]
> 0	Reference (0)	Reference (0)	Reference (0)
< -1.28	1.21 (0.10; 2.33)	-4.25 (-32.12; 23.63)	-0.07 (-0.26; 0.12)
-1.28/0	0.85 (0.08; 1.62)	-19.20 (-38.58; 0.17)	0.00 (-0.13; 0.14)
Birth weight (g) ^##^	p = 0.016 *** [n = 3,506]	p = 0.084 *** [n = 3,011]	p = 0.539 *** [n = 1,576]
3,000-3,499	Reference (0)	Reference (0)	Reference (0)
< 2,500	0.93 (-0.39; 2.25)	0.10 (-34.84; 35.04)	-0.05 (-0.27; 0.17)
2,500-2,999	0.84 (0.00; 1.69)	1.05 (-21.07; 23.18)	-0.02 (-0.16; 0.13)
≥ 3,500	-0.19 (-0.97; 0.59)	20.81 (0.93; 40.70)	0.02 (-0.12; 0.15)
Height/length-for-age z-scores - 2 years ^###^	p = 0.003 *** [n = 2,590]	p = 0.4789 ^#^ [n = 2,240]	p = 0.031 ^#^ [n = 1,155]
-1/1	Reference (0)	Reference (0)	Reference (0)
≤ -2	1.90 (0.63; 3.17)	12.07 (-19.47; 43.62)	0.11 (-0.10; 0.32)
-2/-1	1.16 (0.24; 2.08)	14.63 (-8.68; 37.94)	-0.12 (-0.28; 0.04)
≥ 1	0.24 (-1.17; 1.65)	19.77 (-15.72; 55.26)	0.25 (0.00; 0.51)
Height/length-for-age z-scores - 4 years ^###^	p = 0.009 *** [n = 2,541]	p = 0.2345 ^#^ [n = 2,199]	p = 0.024 ^#^ [n = 1,128]
-1/1	Reference (0)	Reference (0)	Reference (0)
≤ -2	2.54 (1.13; 3.95)	14.24 (-20.16; 48.63)	0.08 (-0.15; 0.31)
-2/-1	0.55 (-0.38; 1.48)	1.40 (-21.51; 24.31)	-0.05 (-0.20; 0.11)
≥ 1	0.57 (-0.94; 2.09)	37.18 (-0.57; 74.93)	0.39 (0.12; 0.65)
Weight-for-length/height z-scores - 2 years ^###^	p = 0.466 *** [n = 2,589]	p = 0.202 *** [n = 2,239]	p = 0.185 *** [n = 1,155]
-1/1	Reference (0)	Reference (0)	Reference (0)
≤ -1	-0.27 (-2.08; 1.54)	10.43 (-36.26; 57.12)	-0.02 (-0.35; 0.31)
1/2	-0.09 (-0.96; 0.78)	17.41 (-4.68; 39.50)	0.05 (-0.10; 0.20)
≥ 2	0.78 (-0.67; 2.22)	16.37 (-19.71; 52.45)	0.18 (-0.08; 0.43)
Weight-for-length/height z-scores - 4 years ^###^	p = 0.003 *** [n = 2,539]	p = 0.039 *** [n = 2,198]	p = 0.004 *** [n = 1,128)
-1/1	Reference (0)	Reference (0)	Reference (0)
≤ -1	0.11 (-1.67; 1.89)	-0.63 (-47.44; 46.18)	0.03 (-0.27; 0.34)
1/2	0.99 (0.09; 1.90)	20.51 (-2.51; 43.54)	0.07 (-0.08; 0.23)
≥ 2	2.05 (0.55; 3.55)	27.73 (-7.51; 66.98)	0.43 (0.19; 0.68)

95%CI: 95% confidence interval; BMI: body mass index.

* Analyses of association with mean arterial pressure were adjusted for height in adulthood;

** Model 1: adjusted for family income, maternal schooling and maternal age at birth, maternal skin color, parity, maternal smoking during pregnancy, pre-pregnancy BMI;

*** p-value for linear trend;

^#^ p-value for heterogeneity;

^##^ Model 2: model 1 + gestational age;

^###^ Model 3: model 1 + birth weight for gestational age + breastfeeding duration.

Triglycerides were higher among subjects who were in the extreme birth weight categories. Height-for-age z-scores at 4 years old was positively associated with triglycerides and C-reactive protein. Triglycerides and C-reactive protein were higher among subjects in the extreme categories of weight-for-height at 4 years of age. Moreover, random blood glucose was higher among subjects whose weight-for-height z-score was ≥ 2 z-score (Table 7).

**Table 7:** Coefficients estimated by multiple linear regression for triglycerides, non-HDL cholesterol, plasma glucose, and C-reactive protein at 30 years, according to birth conditions and nutritional status.

	Exponential means (95%CI)		Adjusted regression coefficient (95%CI)	
	Triglycerides (mg/dL)	C-reactive protein (mg/dL)	Non-HDL cholesterol (mg/dL)	Plasma glucose (mg/dL)
Gestational age (weeks) *	p = 0.393 ** [n = 2,842]	p = 0.726 ** [n = 1,888]	p = 0.601 *** [n = 2,842]	p = 0.350 *** [n = 2,844]
> 39	Reference (1)	Reference (1)	Reference (0)	Reference (0)
< 37	0.96 (0.88; 1.04)	1.10 (0.84; 1.44)	-1.85 (-7.45; 3.76)	-2.32 (-5.96; 1.31)
37-38	0.99 (0.95; 1.05)	0.98 (0.85; 1.13)	1.10 (-2.04; 4.23)	0.58 (-1.45; 2.61)
Birth weight according to gestational age (z-score) *	p = 0.548 ** [n = 2,841]	p = 0.996 *** [n = 1,888]	p = 0.634 ** [n = 2,841]	p = 0.316 ** [n = 2,843]
> 0	Reference (1)	Reference (1)	Reference (0)	Reference (0)
< -1.28	1.03 (0.96; 1.10)	1.00 (0.83; 1.19)	1.07 (-2.95; 5.09)	1.00 (-1.60; 3.60)
-1.28/0	0.99 (0.95; 1.04)	1.00 (0.88; 1.14)	0.23 (-2.57; 3.03)	0.99 (-0.82; 2.80)
Birth weight (g) ^#^	p = 0.039 *** [n = 3,524]	p = 0.234 ** (n = 2,347)	p = 0.202 *** [n = 3,524]	p = 0.122 *** [n = 3,527]
3,000-3,499	Reference (1)	Reference (1)	Reference (0)	Reference (0)
< 2,500	1.05 (0.98; 1.13)	1.08 (0.86; 1.36)	-3.86 (-8.76; 1.04)	-1.76 (-5.13; 1.60)
2,500-2,999	1.04 (0.99; 1.10)	1.07 (0.93; 1.23)	1.67 (-1.46; 4.79)	2.01 (-0.14; 4.15)
≥ 3,500	1.06 (1.02; 1.11)	0.99 (0.87; 1.12)	0.27 (-2.60; 3.15)	0.68 (-1.29; 2.64)
Height/length-for-age z-scores - 2 years ^##^	p = 0.279 ** [n = 2,596]	p = 0.792 ** [n = 1,723]	p = 0.640 ** [n = 2,596]	p = 0.114 *** [n = 2,599]
-1/1	Reference (1)	Reference (1)	Reference (0)	Reference (0)
≤ -2	1.00 (0.93; 1.08)	1.00 (0.82; 1.23)	1.65 (-2.92; 6.22)	2.66 (-0.30; 5.61)
-2/-1	0.98 (0.93; 1.04)	1.03 (0.89; 1.20)	0.98 (-2.38; 4.34)	0.78 (-1.39; 2.95)
≥ 1	1.07 (0.99; 1.17)	0.97 (0.76; 1.24)	1.59 (-3.58; 6.76)	3.14 (-0.19; 6.47)
Height/length-for-age z-scores - 4 years ^##^	p = 0.001 ** [n = 2,552]	p = 0.045 ** [n = 1,694]	p = 0.021 *** [n = 2,552]	p = 0.307 *** [n = 2,555]
-1/1	Reference (1)	Reference (1)	Reference (0)	Reference (0)
≤ -2	0.91 (0.85; 0.99)	0.88 (0.71; 1.10)	-0.53 (-5.46; 4.40)	1.01 (-2.27; 4.68)
-2/-1	0.99 (0.94; 1.04)	0.85 (0.73; 0.99)	2.50 (-0.80; 5.81)	0.67 (-1.54; 2.87)
≥ 1	1.15 (1.05; 1.26)	1.06 (0.83; 1.35)	8.02 (2.51; 13.54)	3.40 (0.24; 7.05)
Weight-for-length/height z-scores - 2 years ^##^	p = 0.436 ** [n = 2,595]	p = 0.333 ** [n = 1,723]	p = 0.271 ** [n = 2,595]	p = 0.098 ** [n = 2,598]
-1/1	Reference (1)	Reference (1)	Reference (0)	Reference (0)
≤ -1	0.98 (0.89; 1.09)	1.02 (0.76; 1.36)	0.78 (-5.81; 7.38)	0.20 (-4.07; 4.46)
1/2	1.00 (0.95; 1.05)	1.01 (0.87; 1.16)	1.41 (-1.79; 4.60)	-1.47 (-3.53; 0.60)
≥ 2	1.05 (0.96; 1.14)	1.17 (0.95; 1.45)	2.85 (-2.44; 8.14)	-2.16 (-5.56; 1.25)
Weight-for-length/height z-scores - 4 years ^##^	p = 0.005 ** [n = 2,550]	p = 0.012 ** [n = 1,693]	p = 0.045 ** [n = 2,550]	p = 0.064 ** [n = 2,553]
-1/1	Reference (1)	Reference (1)	Reference (0)	Reference (0)
≤ -1	1.06 (0.96; 1.17)	1.03 (0.76; 1.39)	0.45 (-6.10; 6.99)	-0.45 (-4.81; 3.92)
1/2	1.07 (1.01; 1.12)	1.20 (1.04; 1.39)	4.30 (0.97; 7.63)	0.45 (-1.76; 2.66)
≥ 2	1.14 (1.04; 1.26)	1.23 (0.99; 1.54)	2.67 (-2.71; 8.05)	4.05 (0.48; 7.63)

95%CI: 95% confidence interval; BMI: body mass index.

* Model 1: adjusted for family income, maternal schooling and maternal age at birth, maternal skin color, parity, maternal smoking during pregnancy, pre-pregnancy BMI;

** p-value for linear trend;

*** p-value for heterogeneity;

^#^ Model 2: model 1 + gestational age;

^##^ Model 3: model 1 + birth weight for gestational age + breastfeeding duration.

Table 8 shows that carotid intima-media, pulse wave velocity, and mean arterial pressure were associated with conditional relative weight gain from 2 to 4 years.

**Table 8:** Coefficients estimated by multiple linear regression for mean arterial pressure, carotid intima-media thickness, pulse wave velocity, triglycerides, non-HDL cholesterol, plasma glucose, and C-reactive protein at 30 years, according to conditional growth in childhood.

	Exponential means (95%CI)			Adjusted regression coefficient (95%CI)			
	Mean arterial pressure (mmHg)	Triglycerides (mg/dL)	C-reactive protein (mg/dL)	Carotid intima-media thickness (µm)	Pulse wave velocity (m/s)	Non-HDL cholesterol (mg/dL)	Plasma glucose (mg/dL)
Conditional length at age 0-2 years * (z-score)	p = 0.128 ** [n = 2,590]	p = 0.029 ** [n = 2,596]	p = 0.298 *** [n = 1,723]	p = 0.263 ** [n = 2,240]	p = 0.105 ** [n = 1,155]	p = 0.518 *** [n = 2,596]	p = 0.956 *** [n = 2,599]
≤ 0	Reference (0)	Reference (1)	Reference (1)	Reference (0)	Reference (0)	Reference (0)	Reference (0)
0/1	-0.91 (-1.90; 0.09)	1.01 (0.95; 1.07)	0.87 (0.73; 1.03)	-2.15 (-27.42; 23.12)	0.00 (-0.18; 0.18)	-2.66 (-6.31; 0.99)	-0.52 (-2.87; 1.84)
1/2	-0.43 (-1.52; 0.66)	1.03 (0.97; 1.10)	0.99 (0.84; 1.18)	15.60 (-11.54; 42.74)	0.06 (-0.12; 0.24)	0.14 (-3.80; 4.09)	0.30 (-2.27; 2.86)
≥ 2	-0.87 (-2.02; 0.28)	1.07 (1.01; 1.15)	1.05 (0.87; 1.27)	11.55 (-16.62; 39.73)	0.16 (-0.03; 0.35)	-0.59 (-4.68; 3.50)	-0.21 (-2.85; 2.43)
Conditional relative weight at age 0-2 years ^#^ (z-score)	p = 0.008 *** [n = 2,589]	p = 0.210 *** [n = 2,595]	p = 0.107 *** [n = 1,723]	p = 0.083 ** [n = 2,239]	p = 0.126 *** [n = 1,155]	p = 0.422 *** [n = 2,595]	p = 0.125 ** [n = 2,598]
≤ 0	Reference (0)	Reference (1)	Reference (1)	Reference (0)	Reference (0)	Reference (0)	Reference (0)
0/1	-0.98 (-1.96; 0.00)	0.96 (0.91; 1.02)	0.87 (0.74; 1.03)	9.57 (-15.89; 35.03)	-0.11 (-0.28; 0.07)	-2.44 (-6.12; 1.24)	-1.30 (-3.68; 1.08)
1/2	-0.80 (-1.89; 0.28)	0.97 (0.92; 1.03)	0.92 (0.77; 1.11)	17.78 (-9.67; 45.23)	-0.05 (-0.24; 0.14)	-0.74 (-4.74; 3.25)	-1.79 (-4.37; 0.79)
≥ 2	1.07 (0.02; 2.16)	1.04 (0.97; 1.11)	1.11 (0.94; 1.32)	20.48 (-7.11; 48.08)	0.15 (-0.03; 0.34)	1.41 (-2.59; 5.41)	-1.51 (-4.09; 1.07)
Conditional height at age 2-4 years ^##^ (z-score)	p = 0.063 *** [n = 2,405]	p = 0.027 ** [n = 2,413]	p = 0.028 ** [n = 1,601]	p = 0.216 ** [n = 2,078]	p = 0.103 *** [n = 1,070]	p = 0.206 ** [n = 2,413]	p = 0.302 ** [n = 2,416]
≤ 0	Reference (0)	Reference (1)	Reference (1)	Reference (0)	Reference (0)	Reference (0)	Reference (0)
0/1	-0.97 (-2.00; 0.06)	1.01 (0.96; 1.08)	0.92 (0.78; 1.08)	-0.24 (-26.48;25.99)	-0.03 (-0.21; 0.15)	1.63 (-2.13; 5.39)	1.73 (-0.74; 4.21)
1/2	0.04 (-1.12; 1.19)	1.06 (0.99; 1.13)	1.11 (0.92; 1.32)	11.54 (-17.60; 40.68)	-0.16 (-0.36; 0.05)	0.25 (-3.92; 4.41)	0.26 (-2.48; 3.00)
≥ 2	0.80 (-0.35; 1.94)	1.06 (1.00; 1.13)	1.22 (1.01; 1.47)	16.49 (-12.10; 45.08)	0.15 (-0.04; 0.33)	3.09 (-1.02; 7.20)	1.58 (-1.13; 4.28)
Conditional relative weight at age 2-4 years ^###^ (z-score)	p < 0.001 ** [n = 2,403]	p = 0.001 ** [n = 2,411]	p = 0.001 ** [n = 1,600]	p = 0.010 ** [n = 2,077]	p = 0.015 ** [n = 1,070]	p = 0.002 ** [n = 2,411]	p = 0.003 ** [n = 2,414]
≤ 0	Reference (0)	Reference (1)	Reference (1)	Reference (0)	Reference (0)	Reference (0)	Reference (0)
0/1	0.99 (0.01; 1.98)	1.02 (0.97; 1.08)	1.23 (1.05; 1.44)	18.35 (-6.97; 43.67)	0.09 (-0.08; 0.26)	0.64 (-3.00; 4.27)	0.74 (-1.65; 3.14)
1/2	1.77 (0.60; 2.93)	1.09 (1.01; 1.16)	1.12 (0.92; 1.36)	8.99 (-21.41; 39.40)	0.10 (-0.10; 0.31)	4.83 (0.50; 9.16)	0.76 (-2.09; 3.61)
≥ 2	2.26 (1.07; 3.45)	1.11 (1.03; 1.19)	1.37 (1.15; 1.65)	43.16 (12.70; 73.63)	0.25 (0.05; 0.45)	6.15 (1.80; 10.51)	4.88 (2.02; 7.74)

95%CI: 95% confidence interval; BMI: body mass index.

* Model 3: adjusted for family income, maternal schooling and maternal age at birth, maternal skin color, parity, maternal smoking during pregnancy, pre-pregnancy BMI + birth weight for gestational age + breastfeeding duration;

** p-value for linear trend;

*** p-value for heterogeneity;

^#^ Model 4: model 3+ conditional length 0-2;

^##^ Model 5: model 4+ conditional weight 0-2;

^###^ Model 6: model 5 + conditional height 2-4.

Conditional length and relative weight gain from birth to 2 years of age were not associated with non-HDL cholesterol, glucose, and C-reactive protein. On the other hand, linear growth from 2 to 4 years was positively associated with triglycerides and C-reactive protein, whereas conditional relative weight gain from the same period showed a positive association with triglycerides, non-HDL cholesterol, plasma glucose, and C-reactive protein.

Table 9 shows that BMI at 30 years captured the total effect of relative weight gain from 2 to 4 years old on carotid intima-media thickness, triglycerides, non-HDL cholesterol, C-reactive protein, and almost all the association with mean arterial pressure, pulse wave velocity, and plasma glucose. As previously mentioned, the interaction between conditional relative weight at 4 years of age and BMI at adulthood was evaluated. We observed no evidence of effect modification (p-value for interaction > 0.1).

**Table 9:** Total effect, natural direct effect, natural indirect effect, and proportion of mediation * (via body mass index - BMI - at 30 years) of conditional relative weight at age 2-4 on triglycerides, non-HDL cholesterol, plasma glucose, C-reactive protein, mean arterial pressure, carotid intima-media thickness, and pulse wave velocity.

Outcome	Total effect β (95%CI)	Natural direct effect β (95%CI)	Natural indirect effect β (95%CI)	Proportion mediated (%)
Mean arterial pressure (mmHg) [n = 2,301]	0.84 (0.45; 1.23)	0.12 (-0.30; 0.54)	0.72 (0.46; 0.98)	85
Carotid intima-media thickness (µm) [n = 2,301]	10.38 (0.33; 20.42)	-2.72 (-13.64; 8.20)	13.09 (8.18; 18.01)	100
Pulse wave velocity (m/s)	0.08 (0.01; 0.14)	0.02 (-0.05; 0.08)	0.06 (0.03; 0.09)	80
Triglycerides (mg/dL) [n = 2,301]	0.03 (0.01; 0.05)	-0.01 (-0.04; 0.01)	0.04 (0.03; 0.06)	100
Non-HDL cholesterol (mg/dL) [n = 2,301]	1.80 (0.36; 3.23)	-0.51 (-2.02; 0.99)	2.31 (1.49;3.13)	100
Plasma glucose (mg/dL) [n = 2,301]	1.16 (0.08; 2.25)	0.19 (-0.80; 1.19)	0.97 (0.46; 1.48)	83
C-reactive protein (mg/dL) [n = 2,301]	0.09 (0.03; 0.14)	-0.04 (-0.10; 0.03)	0.12 (0.08; 0.16)	100

95%CI: 95% confidence interval.

Note: Mediator: BMI at age 30; Base confounders: family income, maternal schooling and maternal age at birth, maternal skin color, parity, maternal smoking during pregnancy, pre-pregnancy BMI, birth weight for gestational age, breastfeeding duration, conditional length 0-2, conditional weight 0-2, conditional height 2-4; Post-confounders: family income at last visit.

* G-formula.

## Discussion

This study aimed to assess the association of birth conditions and early growth with metabolic cardiovascular risk factors at age 30. Birth weight was negatively associated with mean arterial pressure. Regarding nutritional status in childhood, length/height in childhood was negatively associated with mean arterial pressure, whereas height-for-age z-score at 4 years old was positively associated with triglycerides, non-HDL cholesterol, and C-reactive protein. Weight-for-height at 2 years of age was not associated with the evaluated outcomes; however, at 4 years of age, weight-for-height was positively related to blood pressure, carotid intima-media, pulse wave velocity, triglycerides, and C-reactive protein. Concerning the association with early growth, poor linear growth during the first two years of life was associated with lower triglycerides, whereas linear growth from 2 to 4 years old was positively associated with triglycerides and C-reactive protein. Relative weight gain in childhood, despite the age, was positively related to mean arterial pressure. In late childhood, relative weight gain was positively associated with carotid intima-media thickness, pulse wave velocity, triglycerides, non-HDL cholesterol, plasma glucose, and C-reactive protein. Mediation analysis showed that BMI in adulthood is an important mediator of the association of relative weight gain in childhood with cardiometabolic risk factors in early adulthood.

After controlling for confounders, birth weight was negatively associated with mean arterial pressure. A meta-analysis including 53 studies reported that the odds of hypertension was 30% (odds ratio = 1.30; 95% confidence interval: 1.16; 1.46) higher among low birth weight subjects. Since a negative association was also observed with birth weight according to gestational age - whereas gestational age was not associated with blood pressure - the observed association with blood pressure is probably due to intrauterine growth restriction. Several mechanisms would explain the association of intrauterine growth with hypertension, including chronic kidney disease and endothelial, vascular, and metabolic abnormalities ^20^ ^,^ ^21^.

Regarding nutritional status in childhood, stunting at 2 and 4 years and poor linear growth during the first two years of life were associated with higher mean arterial pressure. On the other hand, height-for-age z-score at 4 years of age showed a positive association with triglycerides, non-HDL cholesterol, and C-reactive protein. Triglycerides and C-reactive protein were higher among subjects who presented accelerated growth from 2 to 4 years. A positive association of height-for-age z-score and linear growth during childhood with blood pressure has been previously reported ^11^ ^,^ ^13^ ^,^ ^22^ ^,^ ^23^ ^,^ ^24^. Notably, the magnitude of association of linear growth with blood pressure decreased in some cases after controlling for adolescent or adult body size ^13^ ^,^ ^22^. Regarding blood lipids, Cheng et al. ^24^ reported that height gain in childhood was negatively related with HDL and positively with triglycerides at 17.5 years. In the Vellore cohort ^13^, faster linear growth between birth and 3 months of age was positively associated with total cholesterol and triglyceride in men and higher blood pressure in women. For linear growth from 3 months to 6.5 years of age, a positive association with blood pressure, and diastolic blood pressure was observed among women. Linear growth from 6.5 to 15 years of age was positively associated with blood pressure in both sexes, whereas an association with total cholesterol, and triglycerides was only reported in men. Positive associations between height or height gain and blood pressure, cholesterol, and triglycerides were less consistent and mostly explained by adult size, although the associations of blood pressure with linear growth 6.5-15 years of age, and of cholesterol with linear growth 0-3 months of age and 6.5-15 years of age in men remained statistically significant after adjustment for adult size ^13^.

Concerning the negative consequences of relative weight gain in childhood, our findings are consistent with previous studies that have reported that faster relative weight gain, mainly in late childhood, is associated with the development of metabolic cardiovascular risk factors at adolescence and adulthood ^8^ ^,^ ^11^ ^,^ ^13^ ^,^ ^24^ ^,^ ^25^, and this association could be due to the association of weight gain in childhood with fat mass and central adiposity in adulthood ^12^ ^,^ ^25^ ^,^ ^26^. Findings from five low- and middle-income countries suggest that weight trajectories in the first two years of life are more strongly associated with adult lean mass than with fat mass, while weight gain from 2 to 4 years of age is related to fat mass ^12^. Araújo de França et al. ^27^ investigated in the Pelotas cohort the association of size at birth, linear growth, and relative weight gain from birth to adulthood with visceral and subcutaneous abdominal fat thickness at 30 years of age. The study showed that conditional relative weight gain beyond 2 years of age was positively associated with visceral and subcutaneous abdominal fat thicknesses at 30 years. Our mediation analyses showed that BMI at adulthood explained the association of relative weight gain in late childhood with metabolic cardiovascular risk factors.

This study has several strengths, such as a large sample size and anthropometric measurements that were conducted at different time points during childhood. The cohort has been prospectively followed since birth and anthropometric measurements have been performed by a trained staff using standardized methods, reducing possible measurement errors. Moreover, the conditional growth analysis allowed us to examine the independent effects of weight and height gains at different age periods. Conditional variables are uncorrelated and expressing them as z-scores allow for a direct comparison of coefficients within regression models. To our knowledge, this is the first study to assess the mediating role of contemporary nutritional status.

Linear growth during early childhood is positively associated with human capital in adulthood and reduces morbidity and mortality risk in late childhood ^7^ ^,^ ^8^ ^,^ ^9^. Our findings show that early linear growth is not related to most metabolic risk factors. On the other hand, faster relative weight gain in late childhood was associated with cardiometabolic risk factors, in line with previous studies. This association was mediated by BMI at adulthood. Our data support the current focus on promoting improved nutrition and linear growth during the first 1,000 days of life and reinforce the importance of preventing rapid relative weight gain after 2 years of age. We emphasize the need for programs to control excess weight for the prevention of cardiovascular diseases to reduce morbidity and mortality in adult life.
