# Supplementary material for: Birth conditions nutritional status in childhood associated with cardiometabolic risk factors at 30 years of age: a cohort study
Source: Cad Saude Publica. 2023 Jun 26;39(6):e00215522. doi: 10.1590/0102-311XEN215522 (PMC10494699; doi:10.1590/0102-311XEN215522)
Supplement: Supplementary file 1 [file 1678-4464-csp-39-06-EN215522-s.pdf]

Supplementary material. Residual plots from the fitted models.

	Mean Arterial Pressure† (mmHg)	Carotid Intima Media Thickness (µm)	Pulse Wave Velocity (m/s)
**Gestational age[a]**	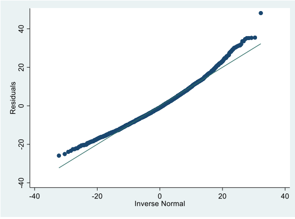	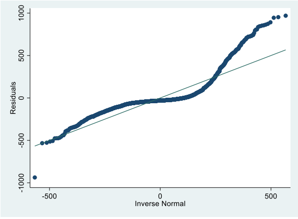	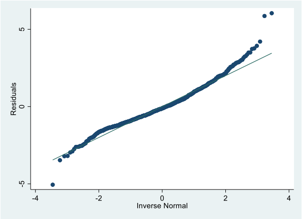
**[illegible] weight according to gestational age[a]**	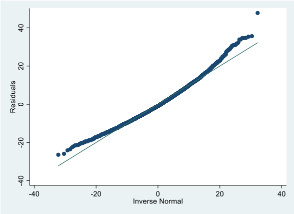	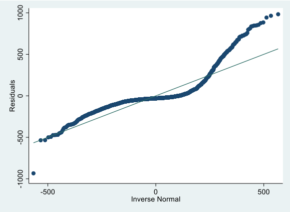	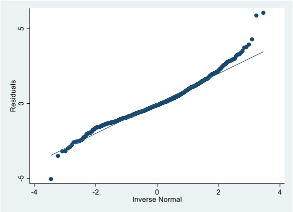
**Birth weight [b]**	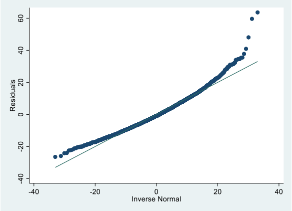	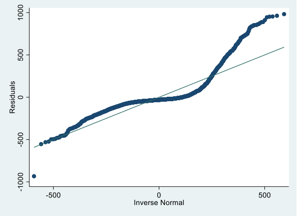	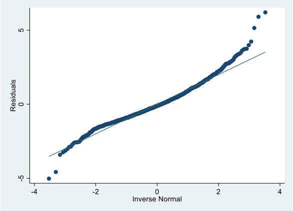
**HAZ 2 years [c]**	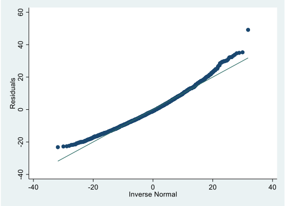	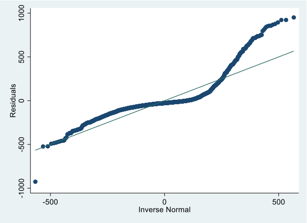	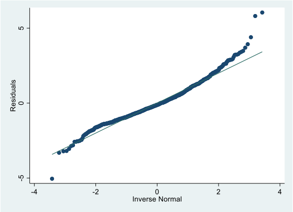
**HAZ 4 years[c]**	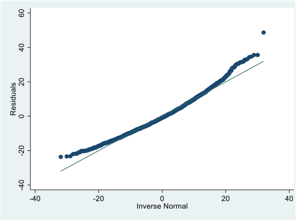	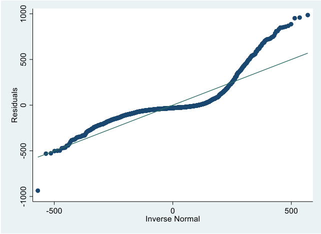	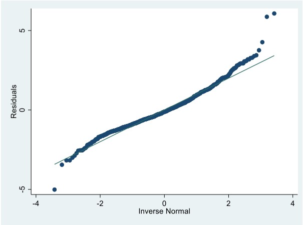
**WHZ 2 years [c]**	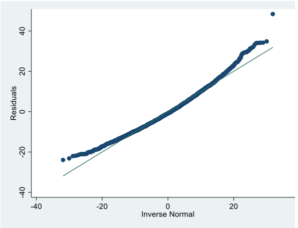	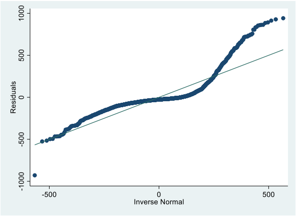	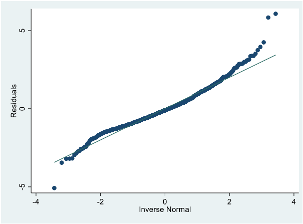
**WHZ 4 years [c]**	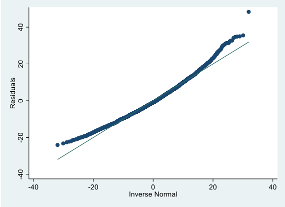	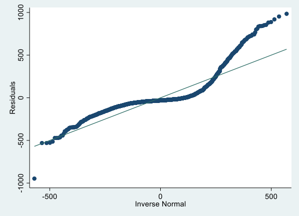	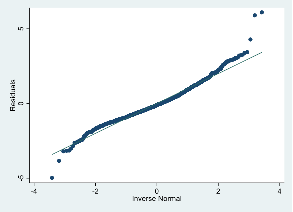

Supplementary material. Residual plots from the fitted models.

	Triglycerides‡ (mg/dl)	Non- HDL-Cholesterol (mg/dl)	Plasma Glucose (mg/dl)	C-Reactive Protein‡ (mg/dl)
**Gestational age[a]**	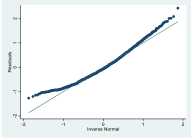	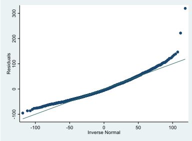	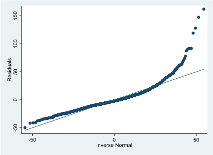	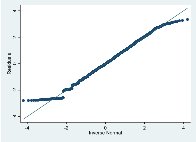
**Birth weight according to gestational age[a]**	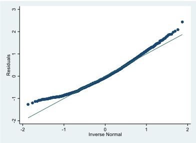	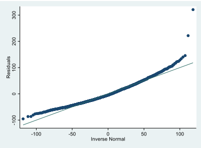	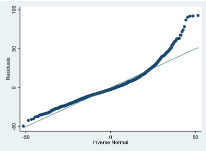	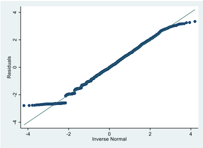
**Birth weight [b]**	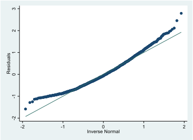	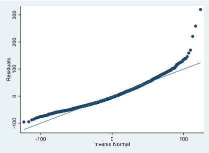	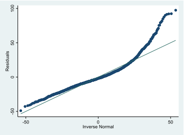	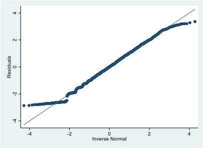
**HAZ 2 years [c]**	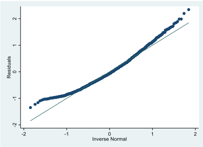	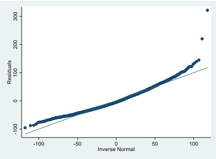	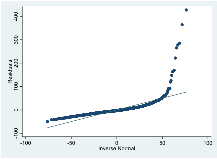	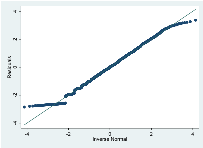
**HAZ 4 years[c]**	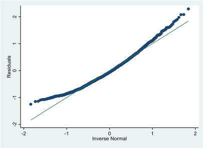	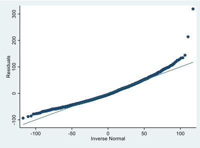	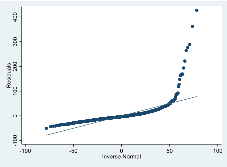	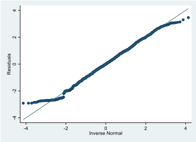
**WHZ 2 years [c]**	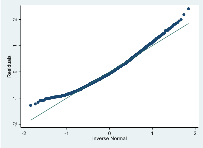	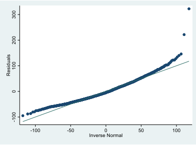	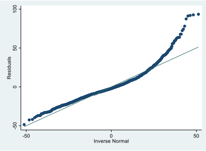	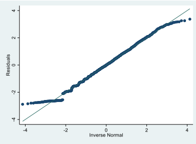
**WHZ 4 years [c]**	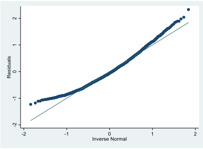	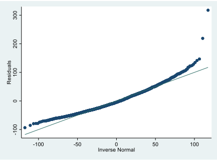	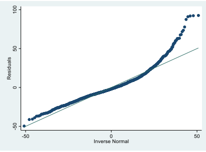	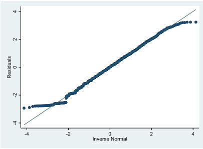

Supplementary material. Residual plots from the fitted models.

	Mean Arterial Pressure† (mmHg)	Carotid Intima Media Thickness (µm)	Pulse Wave Velocity (m/s)	Triglycerides ‡ (mg/dl)	Non- HDL-Cholesterol (mg/dl)	Plasma Glucose (mg/dl)	C-Reactive Protein‡ (mg/dl)
**Conditional length at age 0-2 [c]**	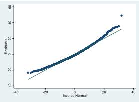	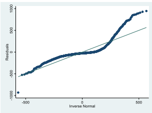	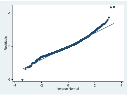	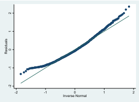	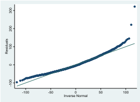	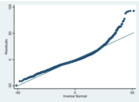	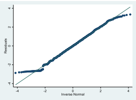
**Conditional relative weight at age 0-2 [d]**	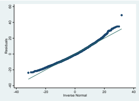	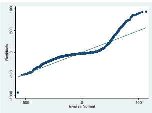	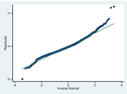	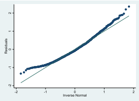	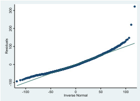	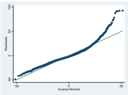	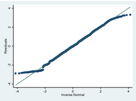
**Conditional height at age 2-4 [e]**	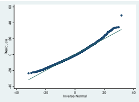	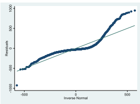	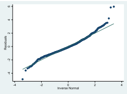	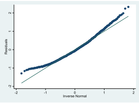	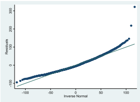	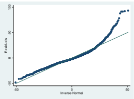	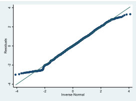
**Conditional relative weight at age 2- 4 [f]**	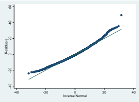	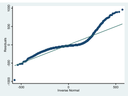	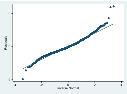	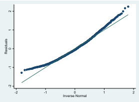	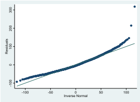	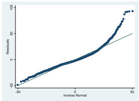	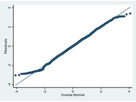
